# Energy Sensors in Female and Male Reproduction and Fertility

**DOI:** 10.1155/2018/8285793

**Published:** 2018-09-27

**Authors:** Joëlle Dupont, Michael J. Bertoldo, Agnieszka Rak

**Affiliations:** ^1^INRA, UMR 85 Physiologie de la Reproduction et des Comportements, F-37380 Nouzilly, France; ^2^Fertility and Research Centre, School of Women's & Children's Health, University of New South Wales, Sydney, NSW 2052, Australia; ^3^Department of Physiology and Toxicology of Reproduction, Institute of Zoology and Biomedical Research, Jagiellonian University, Krakow, Poland

## 1. Introduction

In mammals and birds, the reproductive system is very sensitive to states of energy deficit. For example, chronic food restriction in prepubertal female rats prevents the normal onset of puberty [1]. In chickens, food restriction decreases body weight, the numbers of yellow preovulatory follicles, and the proportion of atretic yellow follicles and increases the age at onset of lay [2]. In pigs, a negative energy balance and a decrease in body fat result in a reduction in litter size and viability of piglets [3]. In sheep, it is well known that an increase in availability of energy substrates is associated with an increase in prolificacy [4]. Furthermore in human, both clinical and experimental studies reveal the negative consequences of obesity on male and female reproductive function [5]. Obese women also have higher rates of many complications in pregnancy, including gestational diabetes and rates of cesarean delivery [6]. Although the clinical impact of obesity on male and female infertility has been well described, the mechanisms involved that could lead to effective treatment are still being elucidated.

The hypothalamic pituitary gonadal (HPG) axis is central to the reproductive system. Pulsatile gonadotropin-releasing hormone (GnRH) released from the hypothalamus stimulates the secretion of luteinising hormone (LH) and follicle-stimulating hormone (FSH) from the anterior pituitary. In females, FSH promotes follicle maturation and LH regulates ovulation whereas in males, FSH is mainly involved in spermatogenesis and LH stimulates the synthesis and the secretion of testosterone by the testis.

Compelling evidence indicates that common regulatory signals involving energy sensors are implicated in the integrated control of energy balance and reproduction. At the whole animal level, energy sensors play a crucial role to indicate whether energy reserves are abundant (obesity) or poor (in case of food restriction and sports training). These energy sensors can be hormones, kinases, or nutrients.

Some fertility disorders related to abnormal metabolism have highlighted the importance of the role of hormones produced by white adipose tissue (adipokines) in the regulation of mechanisms involved in human and animal reproductive functions [7]. It is well known that white adipose tissue provides energy in the form of fatty acids and glycerol to other organs, especially when dietary intake does not meet requirements. However, adipokines produced by white adipose tissue play an important role in the regulation of the reproductive axis [8]. The most studied and described adipokine in the literature is leptin [9]. For example, leptin has been proved to cooperate with other regulatory signals such as ghrelin (the endogenous ligand of the growth hormone (GH)) in the integrated control of energy balance and reproduction [10]. The major involvement of leptin in the regulation of food intake, adiposity, insulin sensitivity, and reproduction has paved the way for the discovery of new adipokines such as adiponectin, visfatin, chemerin, and apelin [11]. Deregulation of plasma levels of these adipokines has been linked to the onset of pathologies such as obesity, insulin resistance, diabetes (type 2 or gestational), and polycystic ovary syndrome (PCOS) [12–15]. Furthermore, the removal of adipose tissue has been demonstrated in mice to inhibit reproductive functions [20]. Thus, an adequate amount and distribution of white adipose tissue may be essential for the completion of normal gametogenesis and fertility.

Given the tight link between energy metabolism and reproduction, in this special issue, we propose to discuss the role of some energy sensors such as adipokines on the fertility in different species including mammals and birds. Birds have some metabolic and reproductive peculiarities interesting to study the relationship between adipokines, metabolism, and reproduction.

This special issue includes one novel research article and 4 reviews summarized as follows:

## 2. Adipokines in Female Reproductive Tractus

### 2.1. Review Article: “Apelin in Reproductive Physiology and Pathology of Different Species: A Critical Review”

In this review article, P. Kurowska et al. described the structure, expression, and function of apelin and its receptor, APJ. They also reported data concerning ELABELA, an endogenous ligand for APJ recently discovered. The authors summarized the physiological and pathological role of apelin in the hypothalamus-pituitary-gonadal axis. For example, this article summarizes the results of a series of recent studies on the effect of apelin on reproduction pathologies, like polycystic ovary syndrome, endometriosis, and ovarian cancer. Many of these pathologies are still in critical need of therapeutic intervention, and the authors report data showing that apelin could be a target in various reproductive pathologies.

### 2.2. Review Article: “Adiponectin: A New Regulator of Female Reproductive System”

K. Dobrzyn et al. focused their attention on adiponectin that is the most abundant adipokine in plasma. After a brief description of the structure of the hormone and its main receptors, AdipoR1 and AdipoR2, the authors described the role of adiponectin in the hypothalamus-pituitary-ovary axis. Furthermore, they reported new data about the involvement of adiponectin in the embryo development and in the physiology of the placenta in different species including humans.

## 3. Adipokines in Gestational Diabetes Mellitus

### 3.1. Research Article: “A Randomised, Controlled Study of Different Glycaemic Targets during Gestational Diabetes Treatment: Effect on the Level of Adipokines in Cord Blood and ANGPTL4 Expression in Human Umbilical Vein Endothelial Cells”

P. Popova et al. investigated the gene expression of adipokines (leptin, adiponectin, and angiopoietin-like protein 4 (ANGPTL4)) in human umbilical vein endothelial cells (HUVECs) and adipokine concentration in cord blood from women with gestational diabetes mellitus (GDM) depending on glycaemic targets. Three groups of patients were studied: control (*n* = 25) and two groups of GDM—GDM1 (tight glycaemic targets, fasting blood glucose < 5.1 mmol/L and <7.0 mmol/L postprandial, *N* = 20) and GDM2 (less tight glycaemic targets, <5.3 mmol/L and <7.8 mmol/L, respectively, *N* = 21). In HUVECs, they showed that ANGPTL4 expression was decreased in GDM patients (with no difference between GDM1 and GDM2) whereas adiponectin gene expression was similar between control and GDM patients and leptin expression was undetectable. In cord blood, the leptin/adiponectin ratio (LAR) was increased in GDM2 compared to controls and GDM1 and did not differ between GDM1 and controls.

## 4. Adipokines in Male Reproductive Function

### 4.1. Review Article: “Adipokines in Semen: Physiopathology and Effects on Spermatozoa”

Y. Elfassy et al. summarized the current data on the localization in the male genital tract and the role of seven adipokines (leptin, adiponectin, resistin, chemerin, visfatin, vaspin, and progranulin) and other cytokines in male reproductive fertility. This review is interesting because most of the published reviews about the link between adipokines and fertility are focused on females but not on males. More precisely, the authors described the regulation of these adipokines in blood and seminal plasmas in different conditions (infertilities associated or not with obesity) based mainly on *in vivo* studies. Furthermore, they summarized some data obtained mainly from *in vitro* studies about the role of the adipokines in semen parameters (sperm concentration, motility, and morphology). Several human polymorphisms of these adipokines are also reported. A list of transgenic animals for different adipokines or adipokine receptors is presented with a description of phenotypes.

## 5. Peculiarities of Adipokines during the Interaction between Reproduction and Metabolism in Birds

### 5.1. “Chicken Is a Useful Model to Investigate the Role of Adipokines in Metabolic and Reproductive Diseases”

N. Mellouk et al. provided an overview of the structure and function related to metabolic and reproductive mechanisms of four adipokines (leptin, adiponectin, visfatin, and chemerin) in avian species as compared to mammals. They emphasize and discuss why avian species are an interesting model to study the adipokines.

## 6. Conclusions

In animal and human species, the optimum body condition level is necessary for normal physiological activities including reproductive efficiency. Inadequate nutrition impairs human fertility and reproductive potential in livestock. This special issue reports how the adipokines convey the body metabolic status to the hypothalamic-pituitary-gonadal axis to regulate fertility. It shows that the molecular mechanisms of these adipokines in reproductive tissues are still unclear. Thus, further research investigations need to be undertaken to unveil the exact mechanism of actions and signalling pathway of adipokines in gonadal dynamics and functions which might improve reproductive efficiency.
